# Neurobiological Convergence in SPDs and ADHD: Insights from a Narrative Review

**DOI:** 10.3390/biology15020198

**Published:** 2026-01-21

**Authors:** Daniele Corbo, Laura Clara Grandi

**Affiliations:** 1 Department of Medical and Surgical Specialties, Radiological Sciences and Public Health, University of Brescia, 25123 Brescia, Italy; 2 Department of Biotechnology and Biosciences, University of Milano-Bicocca, 20126 Milano, Italy

**Keywords:** sensory processing disorders, attention deficit hyperactivity disorder, neuroimaging, sensory dysfunctions, sensory system

## Abstract

The sensory system plays a critical role in development, as it enables the processing and integration of internal and external stimuli. Dysfunctions in this system lead to sensory processing disorders (SPDs), which affect approximately 5–13% of children aged 4–6 years, impacting not only sensory responsiveness but also social interaction, emotional regulation, motor coordination, learning, attention, communication, and sleep. Although SPDs have been extensively investigated from molecular to behavioral levels, their underlying neurobiological mechanisms remain debated, and reliable biomarkers are still lacking. Moreover, due to overlapping behavioral manifestations, SPDs are frequently misdiagnosed as attention deficit hyperactivity disorder (ADHD), leading to challenges in accurate diagnosis and treatment planning. This narrative review aims to synthesize current evidence on the neurofunctional and molecular underpinnings of SPDs in relation to ADHD, providing an integrated perspective on their converging and diverging pathways. By comparing neuroimaging and neurophysiological findings across the two conditions, we seek to deepen understanding of their shared mechanisms, clarify diagnostic boundaries, and inform the development of targeted, evidence-based interventions to address a critical gap in the field.

## Simple Summary

People of all ages may experience differences in how they perceive and respond to sensory inputs such as sound, touch, movement, or other stimuli from their body and environment. These variations can be part of a sensory processing disorder (SPD), but they also occur in other neurodevelopmental or mental health conditions. Sensory challenges can affect emotional regulation, attention, learning, and social interaction. Although ADHD has distinct clinical features, many individuals with ADHD also show sensory difficulties, which can resemble those seen in SPD. This review examines how the brain processes sensory information in both conditions, drawing on evidence from neuroimaging and physiological studies. By comparing shared and distinct neural patterns, we aim to refine diagnostic understanding and support more targeted interventions for individuals facing sensory and attentional challenges in daily life.

## 1. Introduction

Sensory information constantly shapes how individuals perceive and interact with their surroundings. Everyday activities, e.g., following a conversation while observing facial expressions and gestures, or recognizing familiar scents, require the brain to integrate multiple sensory inputs into a coherent percept. This process, known as multisensory integration, allows humans to detect, identify, and respond to environmental stimuli more efficiently than when relying on a single sensory modality [1].

The efficiency of multisensory integration depends on the complexity of the stimuli and on attentional mechanisms that guide sensory processing.

Multisensory integration is modulated by bottom-up and top-down factors. Specifically, bottom-up processes establish the initial multisensory representation based on physical stimulus features, while top-down processes dynamically modulate this representation based on one’s own internal state and behavioral goals. The interplay between both shapes the multisensory perception [2].

When the processes underlying multisensory integration and attentional modulation are disrupted, individuals display sensory processing difficulties, characterized by atypical responses to everyday sensory stimuli. These alterations can manifest as either hypersensitivity or hyposensitivity across one or more sensory modalities, reflecting difficulties in the regulation of social behavior, emotion and cognitive functions. This impaired ability to detect, modulate, and organize sensory information is referred to as a Sensory Processing Disorder (SPD) [3]. It is recognized as neurodevelopmental conditions that can persist across the lifespan, with variable severity and heterogeneous presentation. Behind the behavioral phenotype of the condition, evidence from neurophysiological and neuroimaging studies suggests atypical connectivity and modulation within sensory and associative cortical networks.

Alterations in sensory integration processes are not exclusive to pathological states but may also emerge as part of normal aging or in conditions affecting sensory pathways. For instance, reduced efficiency in multisensory processing has been observed in older adults [4], in individuals with depression, and in cases of peripheral sensory injury [5]. These findings suggest that sensory integration depends on finely tuned neural mechanisms whose disruption—whether due to peripheral, central, or developmental factors—can significantly impact daily functioning.

SPDs frequently co-occur with or are misinterpreted as other conditions such as autism spectrum disorder (ASD) and attention deficit hyperactivity disorder (ADHD).

ADHD is a neurodevelopmental disorder characterized by persistent and pervasive patterns of inattention, hyperactivity, and impulsivity, frequently associated with unusual responses to sensory stimulation [6].

This overlap in behavioral symptoms, e.g., inattention, hyperreactivity, and sensory seeking, has led to diagnostic uncertainty [7]. In order to correctly lead the appropriate therapy, it is critical to ensure a correct diagnosis. In line with this, growing evidence indicates that SPDs and ADHD are underpinned by distinct neural and molecular mechanisms, highlighting the need to disentangle their neurobiological signatures to refine diagnosis and guide targeted interventions.

In this review, we examine the relationship between SPDs and ADHD, highlighting particularly the role of sensory dysfunction as a shared feature. While sensory abnormalities are increasingly recognized as part of the ADHD phenotype, they are also central to the clinical presentation of SPDs, raising important questions about their neurobiological overlap. Despite growing interest, the precise neuronal mechanisms underlying sensory dysregulation in both conditions remain incompletely understood. This gap in knowledge contributes to a significant clinical challenge: the frequent misdiagnosis or conflation of SPDs and ADHD, especially in pediatric populations [8,9].

Both disorders can manifest with similar behavioral profiles—such as distractibility, emotional dysregulation, and motor restlessness—and exert comparable impacts on daily functioning across developmental stages. By exploring converging and diverging neurofunctional pathways, this review aims to clarify the distinctions and intersections between SPDs and ADHD, ultimately contributing to more accurate diagnosis and targeted interventions. Moreover, since the ADHD–SPDs association is still unclear, the clinical guidelines for ADHD do not include the assessment of the sensory processing pattern [10]. Nevertheless, because therapeutic interventions depend on an accurate diagnosis, correct identification is essential to ensure appropriate treatment and avoid exacerbating the pathological condition. Therefore, there is an urgent need to identify specific biomarkers and to develop new, targeted diagnostic tools and therapies for both SPDs and ADHD.

Considering that sensory discrimination deficit, i.e., a hallmark of SPD, frequently co-occur with attentional dysfunctions in ADHD [6,11], understanding their shared neural and behavioral mechanisms is critical. This overlap remains poorly characterized, contributing to frequent misdiagnosis and hindering the development of precise interventions [8,9].

This narrative review aims to synthesize current findings to provide an integrated perspective on the similarities between SPDs and ADHD, focusing on converging neurofunctional pathways. The review seeks to deepen our understanding of the complex and often intertwined relationship between these conditions, inform more accurate diagnosis, and guide the development of targeted, evidence-based interventions, addressing a crucial gap in the field.

## 2. Materials and Methods

### 2.1. Study Experimental Design

This narrative review was conducted following a re-adaptation of the PRISMA flow [12]. The research was performed in the period between September 2024 and September 2025.

### 2.2. Inclusion Criteria

This narrative review aimed to include all research studies that met specific criteria; they had to be published in English and available in full-text format, including case reports, original research articles, and reviews (narrative, systematic, and meta-analyses), conducted in both humans and animal models. The inclusion of animal studies was intended to provide insights into underlying neuronal mechanisms that are not fully accessible in human studies, supporting a translational understanding of ADHD and SPD. There were no limitations regarding participant age, publication year, or study design.

### 2.3. Study Selection

**Figure 1:**
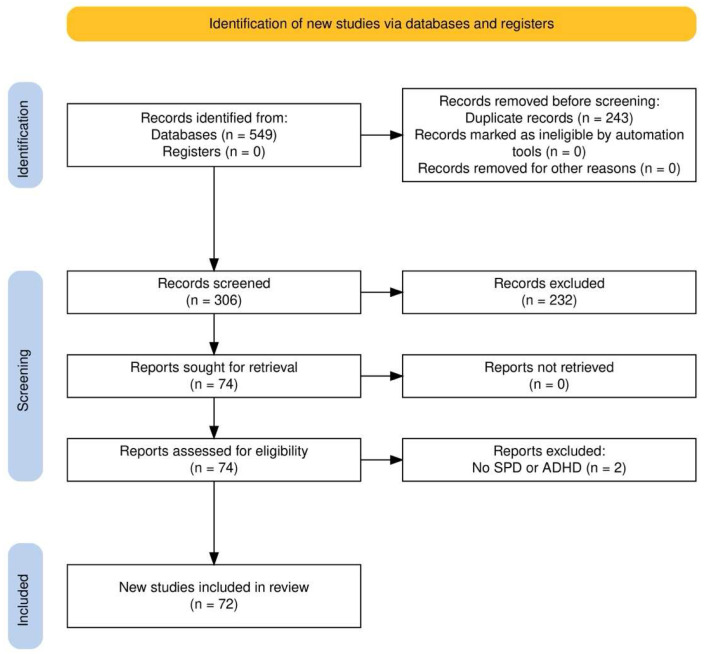
Flowchart of the narrative review process. Starting from 549 studies, 243 records were discarded because of duplicates, 232 were excluded because they partially covered the topic of interest, and 2 further studies were removed after the review of the full text because of missing information, leading to a total of 72 works included in the final narrative review.

To assess the relevant literature in this field, this qualitative review included works published up to September 2025, identified through a search of open-access databases such as PubMed, Google Scholar, Web of Science (WoS), and Scopus. The initial search in all databases utilized a combination of the following terms: [(ADHD OR Attention-deficit hyperactivity disorder) AND (neuroimaging OR fMRI OR MRI) AND (SPD OR Sensory processing disorder)] without specifying a time range of publication dates. In addition to the initially identified articles, we examined for possible inclusion references from those studies, other relevant sources, and literature reviews. Two authors (DC, LCG) conducted the entire selection process, which included choosing databases, applying exclusion criteria, performing secondary searches, and selecting final articles, as illustrated in the flow diagram (Figure 1). The initial search produced 549 results. After removing duplicates, 306 unique articles remained and were screened based on their titles and abstracts. Of these, 232 were excluded for not addressing all key topics. The remaining 74 articles underwent full-text review, and their reference lists were checked for additional relevant studies, though no new articles were identified. A second full-text screening of these 74 articles was conducted to ensure compliance with the inclusion and exclusion criteria, leading to the removal of 2 articles (related to SPD), leading to a total of 72 works included in the final narrative review.

## 3. Results

Recent research has reported that selective disruptions in automatic processing may contribute to ADHD symptoms, potentially linked to atypical sensory processing, which can affect rapid and efficient information processing [13,14]. The presence of sensory deficits in both SPDs and ADHD has been quantified in several studies, providing prevalence data. In detail, up to 64% of children with ADHD present atypical sensory responses, including both over- and under-responsivity [15], in inattentive and hyperactive/impulsive subtypes [16,17]. Conversely, about 40% of children with SPD also meet criteria for ADHD [18]. This bidirectional association has led to the suggestion that SPD could represent a potential clinical indicator within neuropsychiatric disorders such as ADHD [19]. However, current evidence does not support its role as a diagnostic marker, and this remains an open debate in the literature [19]. Similarly, despite individuals with ADHD having a high risk of atypical sensory processing [20], the degree to which the processing is atypical in ADHD remains unclear, and current clinical guidelines for ADHD do not recommend their assessment [10].

Interestingly, early sensory processing is critical for the development and regulation of cortical and subcortical networks that support adaptive sensory function [21]. Indeed, the sensory pathways have hierarchical cortical–subcortical organization that permits the accurate processing of sensory information [21].

To elucidate how these neuronal pathways are involved in sensory deficits, studies have employed somatosensory-evoked potentials, sympathetic indices of nervous system activity (e.g., electrodermal reactivity), electroencephalography (EEG), event-related potentials (ERP), and functional magnetic resonance imaging (fMRI). These techniques allow the quantification of abnormal activity relative to neurotypical controls across development. Moreover, EEG and fMRI are nowadays important and valuable diagnostic tools, since they allow the detection of abnormal activity in comparison to control subjects without ADHD [18,22], both in childhood and in adulthood. Each technique has specific strengths and limitations: ERP provides high temporal resolution (milliseconds), while fMRI provides higher spatial resolution; fMRI is also more challenging in pediatric populations. Moreover, for example, fMRI is not always easy to use with children. The following sections summarize the key findings reported in previous studies using these neuronal physiological methodologies. Importantly, where possible, the information from different techniques could be integrated with each other, and it is also possible to perform simultaneous EEG-fMRI studies (e.g., Ref. [17]).

### 3.1. Shared Brain Networks

Despite differences in large-scale brain networks that are prominent in ADHD, evidence indicates a partial overlap with circuits implicated in SPDs [23]. The executive functions, e.g., attention, and sensory gating, as well as reward processing, and emotions [24], are encoded by fronto-striatal circuits and their impairment in both ADHD and SPD conditions [25,26]. Cupertino and colleagues [25] reported reduced volumes in frontal lobes, striatum, and interconnecting white matter in children and adults with ADHD ([25]; see Section 3.3 for details on this study).

The fronto-striato-thalamic circuit represents a distributed network linking the caudate, putamen, thalamus, supplementary motor area, lateral prefrontal cortex, and parietal lobe. Cortese and colleagues [27] conducted a meta-analysis of neuroimaging studies into ADHD, integrating structural and functional findings. They identified consistent abnormalities in fronto-striatal, cerebellar, and parietal regions, associated with the core ADHD symptoms such as inattention, impulsivity, and hyperactivity. Moreover, a second fronto-striatal circuit seems to be involved in the pathological process of ADHD, i.e., the circuit involving the nucleus accumbens and orbitofrontal cortex [28]. In particular, the nucleus accumbens has been shown to be a central player in the neurobiology of ADHD symptoms, because of its involvement in the dopaminergic system, and as such in reward processing and motivation, which is often impaired in ADHD [29]. Nevertheless, its involvement in SPDs is not evidenced. Amygdala and insula involvement is reported in both ADHD and SPDs, playing roles in sensory–emotional integration and social processing. The amygdala is used in both the evaluation and response to emotionally salient stimuli and it is essential for sensory processing [30,31]. For example, it has been shown to delineate a circuit through which the amygdala exerts top-down influence on early sensory processing via projections to the locus coeruleus, in relation to the olfactory system. The olfactory bulb does not receive any direct projections from the amygdala, therefore it influences early sensory processing through the locus coeruleus. These results could potentially shed light on the mechanisms of the sensory abnormalities [32]. Concerning ADHD, contrasting data are present in the literature, with evidence of its abnormal activation in emotion processing and facial recognition [33,34,35]. Adra and colleagues [36] reported, in adults with ADHD, the positive correlation between both sensory craving and under-responsivity with the volume of the amygdala [36]. However, studies directly examining the role of the amygdala in modulating sensory processing within the broader neural networks of ADHD have not been extensively investigated. Similarly, despite the established role of the amygdala in sensory processing, research on SPDs remains considerably more limited than in ADHD, with no studies to date, to our knowledge, directly investigating amygdala-mediated modulation of sensory inputs within the neural circuits underlying these disorders.

The insula cortex plays a pivotal role in the emotional valence of the stimulus, being also a critical hub of the social brain circuit. Indeed, here the social touch is encoded [37,38,39,40]. Moreover, the insula is anatomically and functionally connected to the amygdala [41,42,43]. Importantly, there is evidence of the involvement of the insula in ADHD. For example, Lopez-Larson [44] reported bilateral reduction in anterior insular gray matter volumes in youths with ADHD, in relation to attention and inhibitory capacity in ADHD. Fateh and colleagues [45] reported that ADHD patients have abnormal insular dynamic functional connectivity among distinct brain regions, i.e., left frontal-mid gyrus, left postcentral gyrus, right of cerebellum crus, thalamus and precuneus [45]. Nevertheless this study does not take into account sensory processing in ADHD. Therefore the results could be considered with regards, for example, to cognitive and executive functioning, working memory, learning problems, anxiety-related symptoms, and social-related functions.

Given the social value of social touch, its sensory component and its processing in the insular cortex, an interesting review is that of Smirni and colleagues [46] that evaluated social touch in neurodevelopmental disorders [46].

Nevertheless, to our knowledge, studies directly assessing insula-mediated modulation of sensory inputs within these neural circuits remain limited. This gap underscores the need for further research to elucidate how insular dysfunction contributes to the atypical sensory processing and network-level abnormalities characteristic of ADHD. Research on SPDs is even less developed, with no studies to date directly investigating insula modulation of sensory processing in these conditions. This gap underscores the need for further research to elucidate how insular dysfunction contributes to atypical sensory processing and network-level abnormalities across neurodevelopmental disorders.

### 3.2. Brain Oscillations

Neural oscillations are rhythmic patterns of neural activity originating from single neurons or from neuronal networks, measured by EEG. At the single-cell level, they appear as fluctuations in the resting potential or as rhythmic action potentials able to induce post-synaptic oscillatory activity. At the network level, synchronized neuronal firing produces macroscopic oscillations, which can be detected through electroencephalography. This oscillatory activity occurs across multiple frequency intervals, conventionally categorized as delta (2–4 Hz), theta (4–8 Hz), alpha (8–13 Hz), beta (13–40 Hz), and gamma (60–90 Hz). Each frequency band has been associated with distinct functions, both in physiological and pathological states [47,48]. Therefore, EEG studies are critical since they provide quantitative measures of neural oscillations with established functional associations.

For example, it has been reported that the motor cortex is one of the main locations of the genesis of beta waves, leading to the hypothesis of its involvement in motor functions [49]. Theta oscillations are associated with different cognitive and physiological functions, such as sleep. In line with this association, theta waves are abnormal in sleep-related diseases, as in the narcolepsy with cataplexy condition [50,51]. Theta waves in the mid-frontal area seem to be associated with cognitive functions [52], being involved in error detection and mental vigilance [53], visual working memory [54,55], and in the dynamic adjustment of behavioral performance [56]. In the posterior area, theta activity is associated with target presentation and goal-directed attention, with a typical lateralization to the contralateral hemisphere in adults without ADHD [56,57].

EEG of children with both SPD and ADHD has showed a reduced midline frontal theta activity [58], as well as a greater-elevated theta power difference between a task state and the resting state than typically developing children [59]. In addition, the theta event-related synchronization (ERS) in children with ADHD is increased compared to that in children without ADHD during visual working memory tasks [60]. Because of this evidence, theta activity is considered a marker of attention abilities captured in real time [61].

Another brain activity that is abnormal in ADHD is alpha activity, despite results not being consistent. Alpha waves have a critical role in cognitive, psychomotor, psycho-emotional and physiological functions. Nowadays, there is still a lack of a robust consensus about the definition and the exact functions.

In addition, the posterior alpha modulation was attenuated in covert spatial attention in both adults with ADHD [62] and children [62,63]. Interestingly, this attenuation appears to exhibit hemispheric lateralization, since the weakened alpha modulation in children with ADHD relative to typically developing controls is present in just one hemisphere, in situations in which attention is directed by social cues. In addition, these alterations in alpha activity have been shown to correlate with the severity of inattentive symptoms [64].

Another brain oscillation implicated in ADHD is the mu rhythm. Ter Huurne and colleagues [65] reported that sensorimotor regions in adults with ADHD fail to exhibit task-related increases in mu rhythm, indicating impaired inhibition of task-irrelevant sensorimotor areas. This aberrant modulation of the mu rhythm may reflect dysregulated motor network activity, contributing to the characteristic hyperactivity and motor disinhibition in ADHD [65]. Despite converging evidence suggesting spatial attention deficits in ADHD, findings regarding the relationship between mu rhythm dynamics and attentional control remain inconsistent, highlighting the need for further investigation into the functional connectivity of sensorimotor and attentional networks in this disorder.

### 3.3. Brain Structure

The study of brain structure, encompassing the physical and morphological organization of the brain, provides essential evidence for distinguishing between physiological and non-physiological states. Such analyses help clarify whether and how deficits in specific functions during pathological states relate to distinct brain structures compared to physiological conditions. In this paragraph, we present evidence about the difference between the brains of individuals with SPD and ADHD, in terms of structural organization and anatomical characteristics. While magnetic resonance imaging (MRI) is the most frequently used technique for the study of the brain structure, T1-weighted anatomical imaging is used to highlight the brain’s macrostructural features and diffusion tensor imaging is used to assess the brain’s microstructural organization [66]. Together, these imaging approaches provide complementary information, linking structural alterations to functional and behavioral profiles observed in SPD and ADHD.

A substantial body of research has established that individuals with ADHD exhibit notable structural brain differences compared to neurotypical controls. In this section, we summarize and discuss these studies, highlighting key findings on structural and anatomical alterations. Neuroanatomical investigations have consistently reported reductions in total gray matter volume, as well as decreased volumes in the basal ganglia and cerebellum. Additionally, abnormalities in cortical thickness have been observed within the cerebellum, alongside diminished volume and cortical thickness in both the frontal and temporal lobes. Further volumetric alterations have been identified in limbic regions such as the amygdala and insula. These brain areas are critically involved in executive functioning and emotional regulation, highlighting the neurobiological underpinnings of the behavioral symptoms commonly associated with ADHD. Nevertheless, these findings are not consistent across all studies, maybe because the presence of comorbid disorders in the studied ADHD samples [67,68]. Surprisingly, most studies on the neuroanatomical correlations of ADHD did not investigate or report on the presence of comorbidities, and the few studies in ADHD-only samples were less likely to find volumetric abnormalities in the frontal cortex than studies that included comorbid individuals [68], as well as no volumetric abnormalities in the amygdala [67].

Mark and colleagues [11] found disrupted white matter microstructure in males with both SPD and ADHD. Specifically, they identified a decreased Neurite Density Index (NDI), in (1) projection tracts, having a role in higher-order sensory functions including multisensory integration; (2) the retrolenticular limb of the internal capsule, having the somatosensory and auditory radiations; and (3) the posterior limb of the internal capsule and the cerebral peduncles, containing corticobulbar and corticospinal projection fibers [69].

In addition, commissural fibers of the splenium of the corpus callosum also exhibit a low NDI in males with both SPD and ADHD. Chang et al. [70] observed that white matter in NDI and ODI increases with age during childhood in subjects without SPD or ADHD [70]. Therefore, the delay and/or disruption in its maturation suggests an association between the degree of sensory tract pathology and the emergence of comorbid ADHD [70]. In addition, because of the differences in males and females, the study of Mark and colleagues [11] also highlighted that hypotheses about the mechanism of ADHD in females with SPD require further studies, since there is a small sample size of females with both ADHD and SPD compared to boys [11].

Adra and colleagues [36] examined the relationships between sensory over-responsivity and intracranial volumes in adults with ADHD, focusing on the basal ganglia, including the caudate nucleus, putamen, globus pallidus, nucleus accumbens, subthalamic nucleus, and substantia nigra. These regions are hypothesized to play a role in SPD, as they are involved in sensory processing [23,71,72,73]. Their results revealed positive associations between the amygdala and both the sensory craving and sensory under-responsivity subscales, as well as between the striatum and sensory under-responsivity. Moreover, a significant inverse correlation was observed between the posterior ventral diencephalon and sensory over-responsivity, alongside a marginally significant negative association with the corresponding subscale. Given that sensory over-responsivity has been proposed to involve alterations in the dopaminergic system, these findings lend support to this hypothesis [36]. Cupertino and colleagues [25] reported on the reduction in the volume in frontal lobes, striatum, and their interconnecting white matter, in both children and adults with ADHD. They analyzed two independent cohorts, i.e., the Dutch NeuroIMAGE cohort of children with an average age of 17.2 years, and the Brazilian IMpACT cohort of adult subjects with ADHD. They reported that a reduction in the volume of bilateral fronto-striatal white matter, adjacent to the orbitofrontal cortex, is age-independent, i.e., it is present both in childhood and in adulthood. These results are of critical importance since they demonstrate that even ADHD has a different clinical profile across the lifespan; also, in terms of the comorbidity profile [74,75], these changes do not reflect brain-related modifications [25].

Diffusion tensor imaging (DTI) is an advanced neuroimaging technique that quantitatively assesses the integrity of white matter fiber tracts, which play a critical role in neural connectivity and communication across brain regions. In a particularly informative study, Cha and colleagues [76] employed tractography, i.e., a method that reconstructs white matter pathways from DTI data, to examine the connectivity profiles of individuals with ADHD. Their results revealed significant disruptions in prefrontal and striatal circuits, highlighting altered structural links between regions involved in cognitive control, reward processing, and behavioral regulation when compared to neurotypical controls.

**Figure 2:**
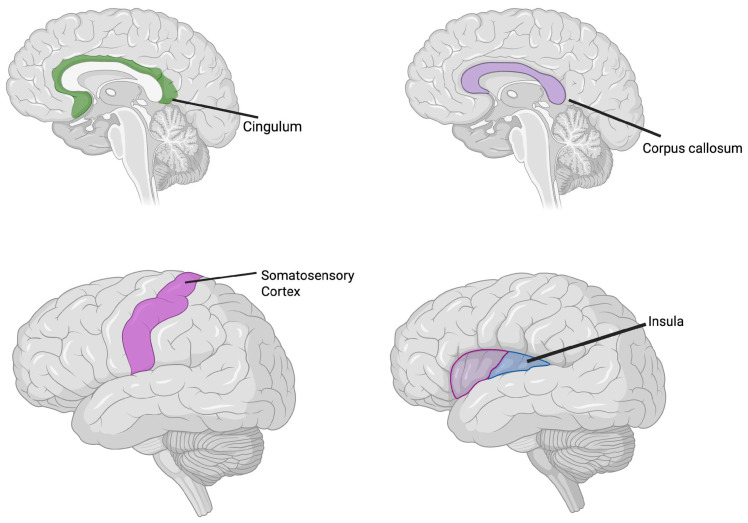
Common brain alteration in ADHD and SPDs. Each panel highlights structural and functional brain regions implicated in ADHD and/or SPD, based on current neuroimaging evidence. Top left (green): The cingulum, involved in attention regulation and cognitive control, shows alterations primarily in ADHD. Top right (purple): The corpus callosum, responsible for interhemispheric communication, is commonly affected in both ADHD and SPD. Bottom left (magenta): The somatosensory cortex, essential for sensory integration and tactile processing, shows atypical activation patterns in individuals with SPD. Bottom right (blue and purple): The insula, a hub for interoception, emotional processing, and social touch, is altered in both disorders. The overlap (purple) indicates shared involvement. Color coding: Green = regions predominantly altered in ADHD. Magenta = regions predominantly altered in SPD. Blue = regions affected in ADHD. Purple = overlapping alterations in both ADHD and SPD. (Created in BioRender. Corbo, D. (2025) https://BioRender.com/lgqx0dz accessed on 14 October 2025).

Additionally, Figure 2 shows the common brain alteration in ADHD and SPDs.

Another structural difference between subjects with and without ADHD is in cortical development. Indeed, it has been reported that maturation progresses in a similar manner regionally in both children with and without ADHD, with primary sensory areas attaining peak cortical thickness before polymodal, high-order association areas. However, there was a marked delay in ADHD in attaining peak thickness throughout most of the cerebrum. The delay is particularly pronounced in prefrontal regions, which are critical for the control of cognitive processes including attention and motor planning [77].

Table 1 summarizes the findings discussed in this narrative review, highlighting the shared features between SPD and ADHD across different domains.

## 4. Discussion

This narrative review aimed to integrate neuroimaging, electrophysiological, and molecular evidence to identify both shared and distinct neurofunctional pathways underlying ADHD and SPDs. Understanding the neural substrates underlying different sensory processing features is essential for identifying potential biomarkers and clarifying overlapping mechanisms between sensory processing disorders (SPDs) and attention deficit hyperactivity disorder (ADHD). An important consideration is that brain structure and function cannot be treated as independent domains, as they are intrinsically interrelated [77,78]. Recognizing this interdependence is crucial for future investigations, as it provides a more comprehensive framework for identifying reliable structural and functional markers across conditions. Such an integrative perspective can ultimately enhance diagnostic precision and deepen understanding of the shared and distinct neurobiological mechanisms underlying SPDs and ADHD.

Although ADHD and SPDs are clinically distinct, converging evidence suggests partial neurobiological overlap, particularly in regions involved in sensory modulation, emotional regulation, and executive control. These shared features may underlie common behavioral manifestations such as distractibility, emotional lability, and sensory over-responsivity.

Electrophysiological studies further illustrate this partial convergence. Altered oscillatory dynamics—particularly in theta and alpha bands—are consistently observed in ADHD and may function as real-time markers of attentional allocation. While SPD-specific electrophysiological data remain limited, existing studies suggest atypical early somatosensory processing and reduced efficiency in cortical modulation. These features converge with ADHD in certain respects but diverge in their timing and functional specificity, reinforcing the idea that the two conditions share some neural vulnerabilities but differ in their core physiological signatures.

Another relevant observation concerns structural variability. Although both ADHD and SPDs present deviations in white matter microstructure and cortical development, the affected regions and their developmental trajectories point to distinct mechanisms. ADHD is characterized by delayed cortical maturation, particularly in prefrontal regions, and reduced volume in basal ganglia structures that support inhibitory control and motivational processes. Conversely, SPDs appear more strongly associated with disruptions in sensory-related projection and commissural tracts, suggesting a more primary alteration in how sensory information travels and is integrated across the brain.

Overall, the present review highlights the importance of adopting a multimodal perspective—encompassing neuroimaging, electrophysiology, and molecular studies—to better capture the complex interplay between sensory processing and attentional control. This integrative perspective offers novel insights into the partially overlapping yet distinct neural mechanisms underlying ADHD and SPDs, with implications for refining diagnostic criteria and developing targeted, evidence-based interventions. Nonetheless, a major limitation remains the scarcity of studies directly comparing the two conditions with their own control groups, as well as research focusing on individuals with ADHD without comorbid SPDs and studies incorporating neurophysiological measures [79,80]. This observation is supported both by our review of the available literature and by previous authors who have similarly highlighted this gap. Therefore, there is a pressing need for multimodal research to elucidate the complex interactions between ADHD and SPDs.

Several limitations should be acknowledged. First, as a narrative rather than systematic review, the present work may be subject to selection bias. Moreover, language and temporal restrictions may have led to the omission of relevant evidence, and the generalizability of the conclusions should therefore be interpreted with caution. Second, the heterogeneity of methodological approaches across studies limits the comparability of findings. Third, few studies control for comorbidities or include well-matched control groups, reducing the generalizability of results. In addition, possible differences related to gender and age should be considered for understanding the potential developmental or sex-specific variations in the manifestation of SPDs and ADHD. Finally, the scarcity of electrophysiological and molecular studies specifically addressing SPDs prevents us from drawing firm conclusions about shared biomarkers with ADHD. These limitations should be considered when interpreting the current findings and designing future research.

## 5. Conclusions

Taken together, these findings underscore the central role of integrative neural mechanisms in shaping sensory processing and multisensory integration in ADHD and sensory processing disorders (SPDs). Future research integrating structural, functional, and electrophysiological approaches will be essential to delineate distinct neurobiological subtypes, establish robust and replicable biomarkers, and guide the development of mechanism-based interventions. From a clinical perspective, understanding these neural mechanisms can directly inform the design of targeted interventions to enhance sensory modulation, attentional control, and cognitive functioning in affected individuals. Advancing such a multidimensional framework holds significant promise for transforming diagnostic precision and therapeutic strategies, ultimately bridging the gap between neural circuitry dysfunction and the sensory–cognitive manifestations observed across neurodevelopmental disorders.

## Figures and Tables

**Table 1:** Comparison between attention deficit hyperactivity disorder (ADHD) and sensory processing disorder (SPD) across prevalence, sensory profile, brain networks, oscillatory activity, structural findings, and cortical development.

Category	ADHD	SPD	Shared/Distinct Features
Prevalence and comorbidity	Up to 64% of children with ADHD show atypical sensory responses, including both over- and under-responsivity.	About 40% of children with SPD also meet criteria for ADHD.	Bidirectional association SPD/ADHD
Sensory profile	Selective deficits in automatic processing and attentional control, influenced by atypical sensory modulation.	Core feature of the disorder.	ADHD shows secondary sensory atypicalities, while SPD shows primary sensory dysfunction.
Brain networks	Fronto-striato-thalamic, fronto-cerebellar, fronto-parietal, and salience networks, with involvement of the nucleus accumbens, amygdala, insula, and prefrontal cortex. Also includes atypical connectivity within the default mode and sensorimotor networks.	Altered connectivity in salience, sensorimotor, and parietal sensory integration networks, involving insula, amygdala, thalamus, and cerebellum. White matter abnormalities affect sensory and interhemispheric tracts.	Shared disruptions in salience, fronto-striatal, and parietal sensory networks, reflecting impaired sensory–emotional integration and attention regulation. ADHD shows broader executive and reward-related circuit alterations; SPD shows more specific sensory–integration network dysfunction.
Brain oscillations (EEG)	Increased theta, decreased alpha, and abnormal mu rhythm modulation.	Reduced frontal theta activity and atypical task-related theta modulation.	Both disorders exhibit atypical theta–alpha oscillatory dynamics reflecting inefficient attentional and sensory processing. Altered sensorimotor (mu) activity further suggests shared disruption in cortical inhibition and neural gating mechanisms.
Brain structure (MRI/DTI)	Reduced gray matter volume in frontal, temporal, cerebellar, and basal ganglia regions; disrupted white matter integrity.	Decreased neurite density in projection and commissural tracts, related to sensory integration.	ADHD shows broader cortical–subcortical alterations; SPD primarily affects sensory integration tracts.
Prefrontal cortex development	Delayed cortical maturation, particularly in prefrontal regions.	No delay reported, but microstructural differences in sensory-related tracts.	ADHD = maturational delay; SPD = sensory-specific microstructural alterations.

## Data Availability

No new data were created or analyzed in this study.
